# Domain-specific introduction to machine learning terminology, pitfalls and opportunities in CRISPR-based gene editing

**DOI:** 10.1093/bib/bbz145

**Published:** 2020-02-02

**Authors:** Aidan R O’Brien, Gaetan Burgio, Denis C Bauer

**Affiliations:** 1 Health and Biosecurity, CSIRO, Sydney, NSW, Australia; 2 John Curtin School of Medical Research, Australian National University, Canberra, ACT, Australia; 3 Department of Biomedical Sciences in the Faculty of Medicine and Health Sciences, Macquarie University, Sydney, NSW, Australia

**Keywords:** machine learning, CRISPR, genome engineering, feature selection, data mining

## Abstract

The use of machine learning (ML) has become prevalent in the genome engineering space, with applications ranging from predicting target site efficiency to forecasting the outcome of repair events. However, jargon and ML-specific accuracy measures have made it hard to assess the validity of individual approaches, potentially leading to misinterpretation of ML results. This review aims to close the gap by discussing ML approaches and pitfalls in the context of CRISPR gene-editing applications. Specifically, we address common considerations, such as algorithm choice, as well as problems, such as overestimating accuracy and data interoperability, by providing tangible examples from the genome-engineering domain. Equipping researchers with the knowledge to effectively use ML to better design gene-editing experiments and predict experimental outcomes will help advance the field more rapidly.

## Introduction

Clustered regularly interspaced short palindromic repeats (CRISPR)-based genome editing has become a popular tool for a range of disciplines, from molecular biology to gene therapy [1, 2]. Driving these applications is the ability of the ‘programmable’ system to target a specific location in the genome through a single guide RNA (sgRNA), which uses Watson–Crick base pairing to locate the genomic target. For example, the CRISPR-associated protein 9 (Cas9) uses this ability to cleave a specific DNA target, resulting in a double-strand break. This in turn can be used to induce arbitrary indels through the error-prone nonhomologous end joining (NHEJ) or microhomology-mediated end joining (MMEJ) DNA repair pathways. Cas9 can also be used to induce specific polymorphisms or insertions using a DNA repair template through the non-error-prone homology-directed repair (HDR) pathway, to modify the function of existing genes or even insert large chunks of DNA. Furthermore, removing the ability of Cas9 to cleave DNA results in the catalytically inactive, dead Cas9 (dCas9), which can be used to be used to control gene expression via transcription and chromatin remodeling [3]. The CRISPR system can also be used for other applications, such as nucleic acid detection [4] or CRISPR-based diagnostics [5], with additional uses reviewed in [6].

Common to all these applications is the demand for control over the involved CRISPR-system functionality; be that binding, cutting or repair, with the aim of maximizing efficiency and specificity. As this is a highly complex interplay of influencing factors—such as target nucleotide sequence, cellular environment and experimental conditions—utilizing a simple rule-based system to choose target sites or design CRISPR sgRNAs has proven to be inadequate [7].

Machine learning (ML) enables capturing this complex interplay of various inputs. Specifically, ML allows researchers to model systems, such as the CRISPR gene-editing technology, for a set of ‘samples’ (experiments) without specifying the relationship between the ‘features’ (target properties or experimental parameters) and the ‘label’ (outcomes of the experiment). In the context of CRISPR modeling, the experimental success, or outcome, can reflect the target site’s ability to generate indels, induce a specific point mutation or control gene expression.

By training on samples, supervised ML algorithms can automatically learn the relationship between features and labels. This representation of the relationship can then be stored in a ‘model’. Subsequently, a model can be used to predict the outcome for ‘unlabelled’ samples, i.e. new samples without a known efficiency. ML hence enables researchers to predict the effectiveness of an sgRNA design *in silico*, rather than having to test every design empirically, saving resources and time.

In addition to predicting the efficiency of sgRNA designs, ML can also be used to predict off-target effects, that is, unintended binding activities to other regions of the genome. While some off-targets can be identified using rules or sequence similarity to find loci with a similar sequence to the target [8, 9], more recent tools are shifting toward the use of ML [10]. This is because sgRNA efficiency for targets, and subsequently off-targets, depends on factors other than sequence similarity. Unlike methods that identify like-sequences in the genome using genomic alignment tools and simply present them as potential off-targets, ML can go one step further and predict the likelihood of these sequences being true off-targets based on efficiency models.

With the potential of ML to predict the editing efficiency and off-target risk of CRISPR/Cas9 experiments, many groups have released tools utilizing ML for this purpose [11]. This review discusses common problems, such as algorithm choice and interpretability, as well as pitfalls, such as overestimating accuracy and data interoperability, to equip researchers employing CRISPR systems for gene editing, with the knowledge to effectively use ML approaches.

### Considerations in data labeling

To train a supervised ML model, one of the first steps is to define an appropriate ‘label’. For CRISPR gene-editing experiments, the label could be cleavage efficiency, knockdown efficiency of a gene or amount of expression measured by fluorescence. This can be represented either ‘discretely’ (e.g. high/low) or ‘continuously’ (e.g. 0 to 1). The representation depends on various factors, such as the algorithm used, the data being modeled and what the desired outcome is. For discrete variables, ‘classification’ algorithms are used, whereas for continuous variables, ‘regression’ algorithms are used.

The sgRNA cleavage efficiency, for example, is continuous; as efficiency is on a range from 0% to 100%. So, given a model trained using a regression algorithm, predicting the efficiencies for four unlabeled sgRNAs would result in each one being assigned a value in this range. For example, [0%, 80%, 90%, 100%]. With higher efficiencies being desired, the clear choice would be an sgRNA prediction of 100%.

Continuous values like efficiency can also be represented discretely. For example, when training a classification model, we can arbitrarily assign ‘< 50%’ efficiency sgRNAs to be ‘low’ and ‘> = 50%’ efficiency sgRNAs to be ‘high’. In this case, the resulting model would classify the previous four targets as [low, high, high, high]. However, this removes the ability to discriminate between the top three targets as now they are all simply ‘high’, rather than 80%, 90% and 100%. Although potentially less informative than regression, classification has the benefit of being faster in training and predicting [12].

**Figure 1:**
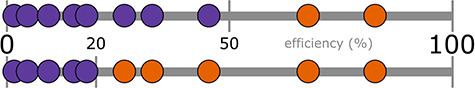
This figure represents two potential decision thresholds (20% and 50%) for 10 hypothetical sgRNA samples (colored dots). Each sample has a DNA cleavage efficiency in the range of 0% and 100%. For a binary classifier, samples above the decision threshold are considered ‘high-efficiency’ (orange) and samples below the decision threshold are considered ‘low-efficiency’ (purple). The decision threshold can be arbitrarily set to any value between 0% and 100%, and an appropriate decision threshold can help keep data balanced. A threshold of 50% in the upper example results in two ‘highs’ and eight ‘lows’. This can result in a poor-performing model as a resulting model could indiscriminately classify all 10 targets as low-efficiency (purple) yet have a relatively good accuracy of 80% (8 out of 10 are correct). However, a threshold of 20% results in five highs and five lows. Now if a model indiscriminately classifies all 10 targets as low-efficiency it will have a more appropriate accuracy of 50% hence being forced to learn the discriminating features between the two classes.

Current regression models for CRISPR efficiency prediction do not achieve a high accuracy, and empirical observations of efficiency are frequently reported to not correlate well with predictions [13]. For example, a target predicted to be 100% efficient may be no more efficient than a target predicted to be 80% efficient. This is because the inherent complexity involved in modeling biological systems can result in models with a limited sensitivity for prediction. Classifying an sgRNA as ‘high’ or ‘low’ efficiency requires less information than placing an sgRNA on a continuous scale of 0% to 100% efficiency. Limited sample sizes and incomplete feature-sets mean that high/low classifications can yield more accurate, albeit less informative, results than continuous predictions (0–100%). Therefore, the ability of classification algorithms to differentiate between highly active sites and others remains valuable in practice, even if only as a stopgap solution until regression algorithms can more accurately model sgRNA efficiency.

A common pitfall with CRISPR data is imbalance [14]. Imbalance is when the positive editing results outnumber the negative results, or vice versa. This can result from researchers only publishing positive results for CRISPR experiments, or from results being overwhelmingly negative due to, for example, the low efficiency of HDR [15, 16]. One way to overcome data imbalance when training a classification model is by choosing an appropriate threshold when converting efficiency from a continuous value to a binary (high/low) value (Figure 1). For example, a threshold of 50% may seem like the obvious choice, but if only 2 out of 10 targets have an efficiency >50%, then a classification model could classify all 10 targets as low efficiency and still have an accuracy of 80%. One potential solution is to adjust the decision threshold [17], for example, from 50% to 20%. This results in an even number of high- and low-efficiency samples. However, now samples with an efficiency of >20% are considered high efficiency, which may not be ideal. A potential solution here is modifying how targets are sampled. For example, rather than choosing targets randomly for training and testing, a bootstrap (sampling with replacement) method can be used to oversample the minority class, as demonstrated by CRISTA and DeepCRISPR [18, 19].

The problem of imbalance is exacerbated for labels with more than two classes. One example is predicting the exact change that results from the editing outcome, as attempted by FORECast [20] and SPROUT [21]. While this greatly increases the control over experimental outcomes, it also increases the number of distinct classes, which in turn requires an increase in training data size to adequately fit the model. For example, for binary labels (high/low) and a perfectly balanced dataset of 1000 samples, each class has 500 (1000/2) samples. If the same dataset is labeled according to the single-nucleotide change (A, C, G or T) present in each sample, the number of samples in each class drops to 250 (1000/4). Predicting other outcomes, such as insertions or deletions will drop the sample size of each class even further, potentially until classes contain only a single sample. To combat this problem and still have enough samples for each of the combinatorial scenarios, FORECast is trained on >40,000 sgRNAs. However, where large sample sizes are not possible, an alternate solution is to limit the number of classes or to train multiple models. For example, rather than having a single model trained on data labeled for every type of change (A, T, AA, AT, TT, etc.), SPROUT relies on multiple models, where one may be trained on length of deletion, and another trained on the type of single-nucleotide change. This allows it to be successfully trained on 1656 sgRNAs.

### Selecting data for a generalizable model

As well as being labeled, each sample must include a set of ‘features’. Features are essentially data (i.e. genetic, epigenetic or experimental) abstracted to a format suitable for training an ML model. The challenge here is to include enough data for an algorithm to produce accurate results, but without including data that is difficult/expensive to obtain, unique to a particular experiment or irrelevant. The aim is to produce a model that can not only make correct predictions but is also ‘generalizable’.

Used in every model mentioned throughout this review are genetic data. This includes the sgRNA sequence, protospacer adjacent motif (PAM) and/or adjacent nucleotides. Although this is primarily because efficient sgRNAs have been demonstrated to prefer certain nucleotides over others [8], a secondary benefit is that sequence information is universal. That is, with the sgRNA sequence being essential for guiding CRISPR/Cas9 to a target, it is a property that will be known for previously conducted CRISPR experiments (resulting in more training data), as well as for experiments in planning. The only variability between the data required for many tools is hence the window size at the sgRNA target (23 nt for ge-CRISPR [22], 26 nt for WU-CRISPR [23] and 30 nt for sgRNA design [24], CRISPRpred [25] and TUSCAN [13]). As each of these tools takes solely sequence information as input, they can predict sgRNA efficiency agnostic to cell type or species.

Azimuth [26] and CRISPRpred [25] aim to improve accuracy over these baseline models by including positional features like ‘exon targeted’ and ‘position of target in gene’. Although Doench *et al.* demonstrated that including these features improves model performance [26], it also has the consequence of decreasing the generalizability compared to sequence-only models. This is because genetic annotation is now required to predict sgRNA efficiency, and this leads to predictions being species-specific. Azimuth will hence fall back to the sequence-only sgRNA design algorithm if positional information is not available.

Chari *et al.* identified epigenetic status to be an additional modulator of efficiency using DNase-seq and H3K4 trimethylation data [27]. While including epigenetic information may have improved the model accuracy, it would make the predictor not only species-specific but also cell type-specific [28]. They hence opted for using only sequence information for their sgRNA scorer and sgRNA scorer 2.0 algorithms [27, 28].

In the pursuit of finding features that add more information and increase accuracy, care should be taken to avoid including as much data as possible, regardless of relevance. Feature sets should ideally include only properties that have a causal relationship to the label. Including irrelevant features (i.e. experiment ID in a tracking system) can be detrimental by increasing the noise and search space, thus potentially reducing model performance [29, 30, 31].

### Translating data to machine-readable features

**Figure 2:**
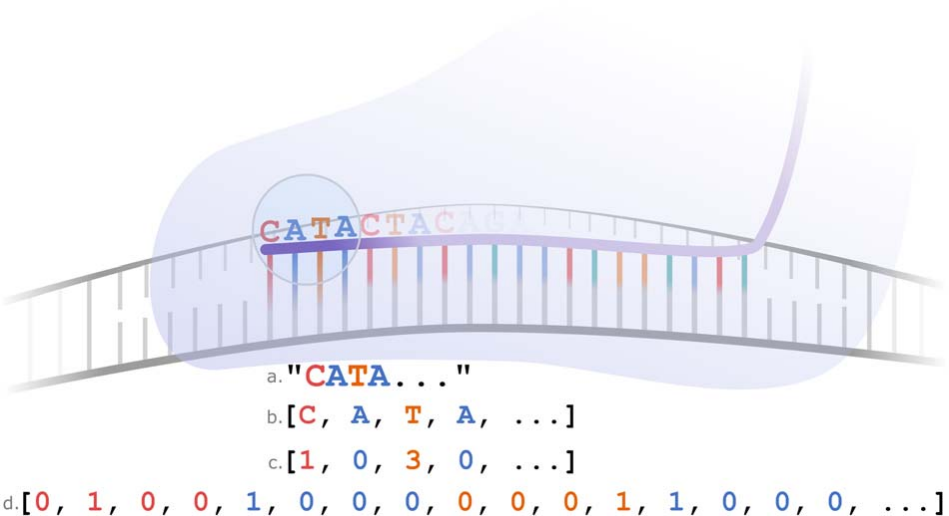
Four different ways to encode the sgRNA nucleotide sequence. Demonstrated are the four encircled nucleotides (CATA): (**A**) as a string—this will not be compatible with many ML algorithms; (**B**) as a list of characters—here, each nucleotide has its own ‘feature’. However, many ML algorithms require features to be represented as numbers; (**C**) as a list of numbers—here, each nucleotide has been arbitrarily assigned a value from 0 to 3. However, algorithms that accept ‘continuous’ features will consider T (3) to be more different from A (0), than T is from G (2) because of the larger difference in the arbitrarily assigned values; (**D**) one-hot encoded—here, each nucleotide is represented as four list elements. One (and only one) of these elements is ‘hot’ (i.e 1) depending on the nucleotide. In this example, the first element being hot, i.e. [1, 0, 0, 0], represents an A. In this representation, all nucleotides are represented as being equally different.

Once data has been identified for inclusion in training, it needs to be processed to meet certain criteria. This is especially true for sequence data because most ML algorithms cannot handle strings natively. For example, an algorithm may be able to identify that ‘CATA’ ‘is’ different to ‘CATT’, but not ‘how’ it is different. To overcome this problem and to capture quantitative differences, sequence features therefore need to be ‘tokenized’ (Figure 2).

Tokenization generally involves breaking down an item, such as a string, into a more generic format, such as an array of numbers. For example, each nucleotide in the DNA (or RNA) alphabet could be represented as a number from 0 to 3 (A = 0, C = 1, G = 2, T = 3). In this case ‘CATA’ would become [1, 0, 3, 0] and ‘CATT’ would become [1, 0, 3, 3]. This is effective because now any ML algorithm can see that only the fourth position has changed. However, this representation is not adequate for algorithms that expect continuous variables because T (3) is more different to A (0) than T (3) is to G (2). Instead, we can process strings further by using ‘one-hot encoding’. This allows nucleotides to be represented as 0s and 1s, by using a separate column for each position in the sequence as well as for each possible nucleotide (Figure 2).

The above processes can be extended to create additional features that represent, for example, nucleotide pairs. This simply entails creating an additional feature column for each permutation and combination of two nucleotides at each position along the sequence. Feature generation can also be driven by domain or expert knowledge. For example, by including a feature to represent the nucleotides either side of the ‘GG’ in the PAM (‘NGGN’) [26], if this were empirically observed to influence efficiency.

### Choosing the right algorithm

With a well-curated feature set and carefully chosen labels, it is possible to train a model. There are many ML algorithms currently used for CRISPR prediction, with each having different advantages and pitfalls. Here, we cover the algorithms frequently used in CRISPR prediction tools. For more detailed comparisons of specific tools, see Chuai *et al.* 2017 [32] and Cui *et al.* [33], and for benchmarks see Yan *et al.* [34].

Two algorithms frequently used in ML are linear regression and logistic regression. While linear regression—used by CRISPRscan [35]—is suited for continuous labels, logistic regression—used by sgRNA design [36]—is suited for discretely labeled data. Both algorithms model linear relationships between the features and label but can also be extended to model nonlinear relationships through nonlinear transformations. For example, Doench *et al.* observed a nonlinear relationship between sgRNA GC content and efficiency, where a high or low guanine-cytosine (GC) content were correlated with a lower activity than a GC content of ~50%. For this nonlinear relationship, they created two disparate features (one for >50% GC and one for below), which enabled the logistic regression algorithm to capture this nonlinear relationship [36]. However, to avoid these manual transformations, algorithms that support nonlinear separation can be used.

One such example that supports nonlinear separation is the support-vector machine (SVM) algorithm. Trained SVM models, which can support classification or regression, are used by sgRNA scorer, sgRNA design, ge-CRISPR, sgRNA scorer 2.0, CRISPRpred, WU-CRISPR, TSAM and CRISPR-DT [27, 36, 22, 28, 25, 23, 37, 38]. SVM algorithms model nonlinear data by transforming features into a high-dimensional representation where linear separation of samples is possible [39]. Although adding these additional dimensions enables SVMs to model the represented data, it obscures which features contributed to the decision process. This lack of transparency, or ‘black box’ behavior, is balanced against explainable models by generally being more accurate [40, 24, 19, 41]. However, this also depends on the algorithm in question, as well as the data being modeled [42, 43, 13].

**Figure 3:**
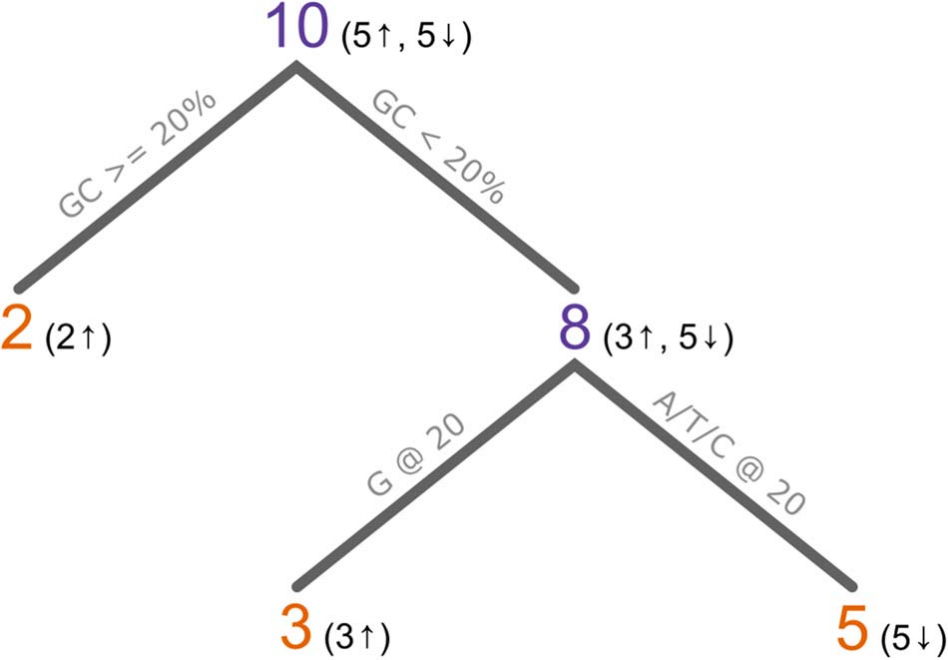
A hypothetical example of a decision tree trained on 10 samples. The first split is on an sgRNA GC content > or <20%, which separates out two samples with a GC content >20%. As both samples are ‘high efficiency’, it results in a pure node (orange). Of the eight sgRNAs with a GC content >20%, three have a high efficiency and five have a low efficiency, so this node is impure (purple). The next split is on the presence (or absence) of a G at position 20 in the sgRNA. All three sgRNAs with a G have a high efficiency and all five sgRNAs without a G have a low efficiency. The resulting nodes are pure, so training concludes. This model would classify new sgRNAs as high if ‘the GC content is >= than 20%’ or ‘the GC content is < 20% and there is a G at position 20’. In reality, such a model would be much more complex with purity not being reached so early, or possibly not at all.

Another important property for the CRISPR space is an algorithm’s ability to capture higher-order interactions between features, i.e. interacting features. In the context of sgRNA efficiency, interacting features are two or more features—be that nucleic, epigenetic or otherwise—that if present together have a correlation with or influence the efficiency. Tree-based methods are one such group of algorithms that can capture higher-order interactions. For example, decision trees model data by iteratively ‘splitting’ the dataset based on features that separate the data. The aim is to generate groups that are ‘pure’, i.e. groups that contain ‘only’ high-efficiency targets or ‘only’ low-efficiency targets. Consider the hypothetical example where sgRNAs with a G at position 20 (G20) ‘and’ a <20% GC content have higher efficiencies than sgRNAs with either or none of these features. The iterative nature of decision trees means that because G20 cannot separate the data into pure groups, a new level is added to split the data based on <20% GC as well (Figure 3). Another benefit of tree-based methods is that they are applicable to both regression and classification [44]. Furthermore, it is possible to interrogate tree-based models to identify which features have the most influence on efficiency prediction, hence making the prediction ‘explainable’.

Two tree-based algorithms used for predicting sgRNA efficiency prediction are random forests (used by CRISPRpred, CRISTA, TUSCAN, CRISPR-DT and CUNE) [18, 13, 25, 45, 38] and gradient boosting (used by Azimuth and SPROUT) [26, 21]. These algorithms are ‘ensemble’ methods, meaning they create models consisting of multiple decision trees. This collection of trees can survey a larger search space and hence are superior to a single tree by improving the generalization or by reducing the error [46].

Recent increases in compute power have enabled another group of ML, deep learning. This group includes algorithms that consist of multiple nonlinear levels, such as convolutional neural networks (CNNs) [47]. Such algorithms have been demonstrated to be successful for image analysis, where the many interconnected levels allow for highly general models that can not only classify images, but also objects within images. The field of sgRNA prediction has recently started using deep learning with the development of tools such as DeepCpf1, DeepCRISPR, off_target_prediction and DeepCas9 [19, 48, 41, 49]. However, deep learning is but one tool in the toolbox and finding the right algorithm remains critical as demonstrated by CRISPR-GNL, a Bayesian ridge regression solution that outperforms its deep learning counterpart, DeepCas9 [50].

However, a unique feature of deep learning is its ability to make preprocessing redundant in some circumstances. Algorithms like CNNs can decompose images containing objects at arbitrary positions/sizes/angles without the need for techniques like cropping, scaling and rotating, not only on the training set but also for novel samples [51]. Being able to uncover underlying patterns in arbitrary data, rather than requiring perfectly curated feature sets, is a useful capability in the CRISPR space.

Currently, the search space for CRISPR targets is much smaller than the typical image analysis task. Where an image may consist of millions of pixels, with objects at arbitrary scales, locations and orientations, a typical CRISPR target consists of 20 to 30 bases, with known coordinates for objects such as the sgRNA target and PAM. Because of this, implementing CRISPR target preprocessing for traditional ML tools is relatively trivial, somewhat negating the need for deep learning algorithms. However, this may change for different Cas enzymes and more complex applications in the future where using the whole genome as input for finding genome-wide optimal targets is conceivable and may improve accuracy.

Hence, choosing the right approach depends on the task (classification versus regression). It also depends on whether the problem is linear or nonlinear, whether feature interactions need to be captured and whether the ability to identify influential features should be present. However, in the case where more than one algorithm is applicable to a problem, comparisons and benchmarks are often appropriate to identify the optimal solution.

### Gaining insights from CRISPR ML models

Training a model on irrelevant features can reduce a model’s performance, but prior to training, it is not always obvious as to which features are relevant. For example, which, if any, epigenetic properties influence sgRNA efficiency and should be included in the model? One way to identify influential features is to selectively train on different subsets of features and subsequently observe variations in the model’s performance as each feature is added. However, training models on different subsets of potentially thousands of features can be inefficient and time-consuming. More appropriate for identifying influential features are explainable models, such as logistic regression and tree-based methods [52, 46]. Such algorithms allow researchers to train a single model on all available features and subsequently rank features by their contribution to the model, or ‘feature importance’.

Feature importance, as well as enabling researchers to only include relevant features, can be extended to ‘hypothesis generation’. While a feature ranking highly is not necessarily indicative of its biological influence over sgRNA efficiency, it can promote the design of further CRISPR experiments to gather support for the generated hypotheses. For instance, features such as position-independent (di) nucleotide count, location of sgRNA within the protein and melting temperatures have been demonstrated to contribute to models and therefore may be involved in DNA cleavage efficiency [26].

### Minimizing the error

Understanding how to interpret the performance metrics of a model is key to ensuring it accurately represents the underlying data. Here we describe ‘bias’ and ‘variance’, and ways in which to minimize them. Commonly, decreasing one (i.e. bias) will come at the cost of increasing the other (i.e. variance) [53]. The aim, therefore, is to find the sweet spot where both forms of error are kept to a minimum.

A model that is overly complex will generally have a high variance, and this can lead to ‘overfitting’. An overfitted model is a model that represents the data it was trained on well (or perfectly), but without being generalizable to the system as a whole (i.e. sgRNA editing efficiency). Because of this, an overfitted model will generally have poor prediction accuracy on samples that it was not trained on. Complexity in a model can arise from noise or outliers in the training data.

Noise can result from features, such as specific nucleotides, that happen to have a relationship to the sgRNA efficiency in the training data, but not in general. Outliers, on the other hand, are samples that are dissimilar from the group they belong to. For example, a negative target (low-efficiency) that happens to have a sequence that is very different from other negative targets, in fact so much so, that it more closely resembles the sequence properties of positive targets (high-efficiency). This may be due to experimental error, especially if the sample size is small, or because the features that would identify it to be a poor target (such as epigenetic information) were not included in the dataset. Training a model on noise and outliers such as these can result in a model with a high variance.

On the other hand, an excessively simplistic model will generally have a high bias. Unlike high-variance models, high-bias models do not capture enough information and tend to underfit. Such models do not accurately model the data they were trained on, let alone the system they were trained to represent. A high-bias model would result from a dataset lacking information, for example if arbitrary sequence data were used, rather than the target sequence, or from an algorithm failing to capture relevant information from the dataset.

To modify how an algorithm learns from the data, with the intent of reducing the bias and variance, its ‘hyperparameters’ can be adjusted. Hyperparameters, unlike model parameters (which are derived through training), are set by the researcher *a priori.* Each algorithm has its own set of hyperparameters, and each hyperparameter modifies a certain aspect of the training process. For example, the random forests algorithm includes hyperparameters such as the ‘number of trees’ and the ‘maximum tree-depth’. Typically, if increasing a hyperparameter increases the model complexity, decreasing it will decrease the model complexity. So, where a deep tree will tend to have a high variance, a shallow tree will typically have a high bias. Therefore, by trialing different hyperparameters, one can find the balance between bias and variance to result in the lowest possible prediction error.

Regardless of hyperparameters and other optimizations, algorithms must have access to a large and representative data set to train accurate models. For CRISPR experiments using the template-free repair pathways, large datasets are now available, with recently published datasets presenting 40 000 Cas9 samples [20] and 15 000 Cas12a samples [48]. However, for other repair pathways (i.e. HDR), there is little data available, impeding the ability to accurately model these biological systems.

## Conclusions

The use of ML in CRISPR applications is improving at a rapid pace, with multiple prediction tools being released every year. Although most models aim to improve the efficacy of CRISPR-Cas9 experiments, they each vary in some detail. While some models are simple and generalizable across organisms and cell types, others are more complex, capturing data like epigenetic information, and are therefore modeling differences in CRISPR efficiency between certain environments. With explainable ML, gaining insights into biological mechanisms becomes more data-driven and encapsulates a wide range of scenarios, reducing the potential for human bias.

With the broad availability of ML-based CRISPR tools, the need to empirically test CRISPR-Cas9 designs to conduct successful experiments is replaced by *in silico* optimization. Here, experiments can be designed algorithmically, optimizing for maximum editing efficiency and minimum off-target effects.

However, available tools have scope for improvements around prediction accuracy or catering for varied experimental parameters, which currently can still lead to misclassification of targets. Also, most current tools are designed for CRISPR-Cas9 NHEJ experiments and are therefore not readily transferrable to other CRISPR systems or DNA repair pathways. In practice, however, a model that performs better than chance is still more economical than a researcher designing guides arbitrarily especially when systematic empirical testing is resource intensive.

Going forward, the following recommendations would enable data scientists and experimental researchers to improve CRISPR ML modeling together:

- Jointly creating large datasets for ML training by submitting results to repositories such as Sequence Read Archive [54] or GenomeCRISPR [55].
- Both positive and negative examples (i.e. target sites that have been shown to be inefficient) are valuable and should be published.
- Computationally identified factors influencing genome editing experiment should be phrased as experimentally testable hypotheses.

## Notes

Key Points

- Machine learning approaches can minimize the empirical testing of potentially inefficient sgRNA designs, reducing the workload involved in CRISPR experiments.
- Current models are not perfect, but advancements can be expedited by researchers jointly creating large datasets and submitting these to repositories such as the Sequence Read Archive.
- Computationally identified factors shown to have a relationship with the efficiency of genome editing experiment should be phrased as experimentally testable hypotheses.

## Notes

**Aidan O’Brien** is a PhD candidate at CSIRO and Australian National University (ANU). His project is on machine learning (ML) for genome engineering. He developed the GT-scan cloud platform now used by 10 000 researchers worldwide.

## Notes

**Gaetan Burgio** is a group leader at the ANU with background in genetics and microbiology. He leads the transgenesis facility at the ANU, and his group develops CRISPR-based gene-editing technologies.

## Notes

**Denis Bauer** is a group leader at CSIRO, with background in bioinformatics and genomics. Her research interests include cloud-based medical genomics, ultra-high dimensional ML and computational genome engineering.
